# Development of a Canadian Food Composition Database of Gluten-Free Products

**DOI:** 10.3390/foods11152215

**Published:** 2022-07-26

**Authors:** Jennifer A. Jamieson, Kelsey Gill, Samantha Fisher, Marcia English

**Affiliations:** Department of Human Nutrition, St. Francis Xavier University, Antigonish, NS B2G 2W5, Canada; gillkelsey05@gmail.com (K.G.); samifisher01@gmail.com (S.F.); menglish@stfx.ca (M.E.)

**Keywords:** gluten-free, food products, food composition database, grains, nutritional quality

## Abstract

Country-specific food composition data are needed for gluten-free (GF) food products to assess nutritional adequacy and diet quality. This research aimed to develop a comprehensive GF food composition database for key GF foods consumed in Canada. Average nutrient data from 167 products were estimated from Nutrition Fact Panel labels and the commercial ingredient list, using an iterative and systematic approach. The database reports mean values for energy and 29 nutrients per 100 g for 33 GF commercial grain-based foods. Nutrient values were evaluated with Health Canada’s nutrient content claims per standard reference serving. On average, GF products were, at minimum, a source of thiamin (73%), riboflavin (70%), niacin (58%), iron (58%), fibre (55%), magnesium (48%), folate (36%), zinc (19%), and calcium (15%). Most GF products were low in saturated fat (85%) and cholesterol (64%) but only 15% were low in total fat and 6% were free of sugar. Micronutrient enrichment and the use of nutrient-dense whole grain flours, legume flours, oil seed husks, and functional fibre ingredients varied within and between categories and brands but appeared to contribute to nutrient content. This database provides a new tool to enhance GF diet assessment in individuals or populations in Canada.

## 1. Introduction

A gluten-free (GF) diet requires the elimination of wheat, rye, and barley grains, as well as processed foods containing these ingredients. A GF diet is indicated in celiac disease, dermatitis herpetiformis, gluten ataxia, non-celiac gluten/wheat sensitivity, and wheat allergy [1]. In Canada, enriched wheat flour is an important contributor of micronutrients and wheat-based staples supply complex carbohydrates, fibre, and phytochemicals [2,3]. Refined wheat flour must be enriched with riboflavin, niacin, folic acid, and iron, whereas vitamin B_6_, pantothenic acid, magnesium, and calcium may be voluntarily added [2]. Food regulations allow for, but do not require, the fortification of GF foods for special dietary use in Canada [2]. Although interest in gluten-free (GF) products has expanded [4], concerns for nutrient inadequacy and diet quality of this restrictive diet remain [5,6]. Historically, GF grain products have relied on starches (e.g., corn, rice, tapioca) as the main ingredients, resulting in foods lower in protein, fibre, and minerals but higher in glycemic index [7]. Although more nutrient-dense ingredients have emerged (e.g., pseudocereals, legume flours, oil seed meals or husks, and functional fibres) to improve the functional, sensory, and nutritional content of GF products, there is a lack of nationally representative food composition data to evaluate dietary intakes. The mandatory Nutrition Fact Panel (NFP) on commercial foods is the primary source of GF nutrient data in Canada but only provides estimates of thirteen core nutrients. Key micronutrients of concern for a GF diet such as folate, zinc, and magnesium [6] are lacking. The primary national food composition database (*Canadian Nutrient File*) contains representative nutrient values for 152 foods, only one of which is presently a commercial GF product [8]. Nutrient data from other national food composition databases are subject to country-specific enrichment or fortification laws and, thus, are not representative of Canadian foods. Previously, a GF food composition database was developed for the Italian diet using an indirect estimation method [9]. Similarly, a team in Spain developed a software application for GF diet evaluation, drawing on the Spanish national food composition database and commercial GF food labels [10]. Given the need for country-specific data and the lack of direct food composition data of Canadian GF foods, we aimed to develop a comprehensive GF nutrient database for commercially available GF grain products consumed in Canada through indirect estimation of the product ingredients. This tool will enhance the dietary assessment of individuals and populations requiring a GF diet in Canada.

## 2. Materials and Methods

Due to a lack of publicly available market share data on GF products and brands, main GF grain sources of nutrients were identified from two regional surveys of dietary intake (240 food records in 2019 [3] and 89 diet recalls in 2017 [11] using a ‘Key Foods’ approach [12] to identify the top food contributors of the 13 core nutrients available on the NFP. Categories of grain-based foods included: breakfast cereals, pasta/noodles, breads, crackers, energy/granola bars, pancakes/waffles, cakes, cookies, and pizza. Bread and cereal categories were sub-divided by enrichment or fortification status and whole grain (defined as first ingredient whole grain) or multi-grain (according to package label) versus refined white products. Loaf breads were separated from English muffins, bagels, and hamburger or hotdog buns. Where possible, at least five commercial brands of each key food were selected for data extraction. Additional brands were derived from a previously published (2016) GF product database (*n* = 3851) [5] and online stores of national grocery retailers in Canada accessed in 2021. Due to limited brand variety of GF foods, categories were fully saturated with 3 to 7 items. Food items containing trace amounts of gluten (e.g., oatmeal) but with well-established nutrient composition data were excluded.

Nutrient data were obtained from NFP of each commercial food product. Missing values were imputed using a previously developed systematic estimation process [9]. Briefly, nutrient data for raw ingredients were obtained from the Canadian Nutrient File (CNF) or US Department of Agriculture (USDA) Food Central databases (per 100 g) and listed in rank order of the ingredient list. Ingredient weights were estimated using known label values (e.g., sugar, salt). Final estimates were then adjusted in an iterative process to match reported macronutrient and energy content on the food label. Missing nutrient values were imputed from the sum of raw ingredients. Estimated nutrient contents were provided for energy (kcal and kJ), water, macronutrients (carbohydrate, total sugar, fibre, protein, cholesterol, total fat, saturated fat, MUFA, PUFA, 18:2 PUFA, 18-3 PUFA), minerals (calcium, iron, magnesium, phosphorus, potassium, sodium, zinc), and vitamins (A, B_1_, B_2_, B_3_, B_6_, B_12_, C, D, total folate, folic acid, food folate). Nutrient values of food additives (e.g., cellulose) were estimated in consultation with a food scientist and informed by published values where possible (see Supplemental Materials: Raw data file). Two ingredients unavailable in CNF/USDA were obtained from ESHA Research Food Research software (Salem, OR, USA). USDA retention factors [13] were applied to nutrient estimations for each food product to account for the loss of nutrients during processing. Cooked noodle values were calculated from dry noodle data using a published 2.7-fold cooking yield for GF spaghetti [14]. For composite foods (e.g., pancakes prepared from a dry mix, vegetable oil, milk, or water), cooking yields [15] were applied to the recipe, while USDA nutrient retention factors were applied per ingredient. Data extraction was performed individually on 167 GF grain products and presented as averages with standard deviations of nutrient values per category (*n* = 12) or sub-category (*n* = 33). Health Canada’s nutrient content claims were applied to average values of fibre, total fat, saturated fat, cholesterol, vitamins, and minerals using standard reference servings for each food category [16].

## 3. Results

### 3.1. Nutrient Content Claims

**Figure 1:**
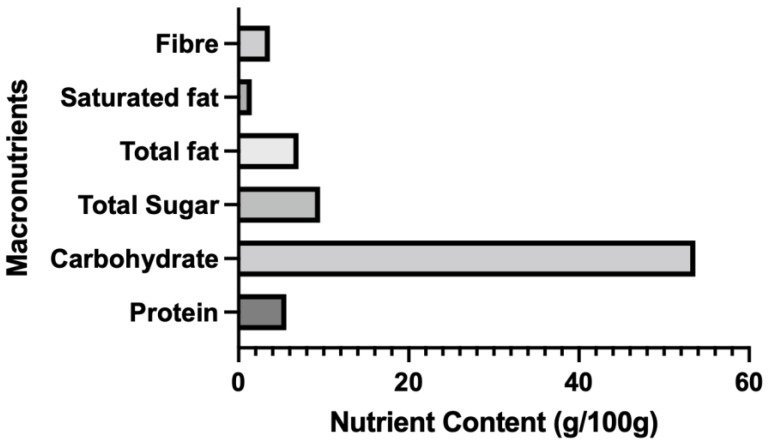
Nutrient content in g/100 g for gluten-free foods across 12 different food categories. The data are presented as mean values.

The macronutrient content (g/100 g) of the gluten-free products evaluated in the present study are shown in Figure 1. On average, GF products in the database were, at minimum, a source of thiamin (73%), riboflavin (70%), niacin (58%), iron (58%), fibre (55%), magnesium (48%), folate (36%), zinc (19%), and calcium (15%). Overall, the products were low in protein content, but carbohydrate content was high. The majority of products were low in saturated fat (85%) and cholesterol (64%), but only 15% were low in total fat and 6% were free of sugar.

### 3.2. Breads and Cereals

Table 1 reports the energy and macronutrient composition of GF breads and cereals. Overall, there was little difference in the energy and macronutrient composition between the bread product categories. GF breads and buns were a high source of dietary fibre, with little apparent variation between white and whole or multi-grain types. This seems to be explained by the addition of ingredients such as psyllium husk and chicory root to refined white GF products. Bagels were a source of fibre, but English muffins were not, likely as a result of reliance on potato, tapioca, and corn starches as main ingredients and the lack of functional fibre additions to English muffins. Sweetened breakfast cereals had the highest total sugar content, and granola cereals had the highest fat content in this category. Fortified cereals and enriched breads tended to be greater sources of calcium, iron (Table S1), and folic acid (Table S2) than unenriched comparative sub-categories. Notably, the B vitamins selected for enrichment varied between brands and products (e.g., some including vitamin B_6_ and niacin but not folic acid or riboflavin). Overall, most breads and cereals were sources of thiamin, riboflavin, niacin, and vitamin B_6,_ regardless of enrichment practices due to the inclusion of psyllium husk powder, brown rice germ and brown rice flour (thiamin), eggs (riboflavin), and whole grain ingredients (niacin, pyridoxal). Whole or multi-grain breads and all cereals were sources of magnesium, phosphorus, and zinc. Overall, sodium content was approximately two- to four-fold higher than potassium content for breads and cereals, with the exception of granola cereal, where dried fruit, nuts, and seeds contributed potassium but less sodium per 100 g than other cereals. The mean sodium content exceeded 400 mg Na/100 g for all breads and cereals except granola (Table S1).

### 3.3. Pastas and Pizzas

Table 2 presents the energy and macronutrient composition of GF pasta or noodles and pizzas. The four categories of pasta/noodles were low in total fat, saturated fat, and cholesterol and had similar energy and nutrient contents, with the exception that buckwheat noodles were a source of fibre. Buckwheat, brown rice, and ancient grain noodles were low in sodium and sources of magnesium, phosphorus, zinc, and vitamin B_6_ (Tables S1 and S2). Buckwheat and ancient grain noodles were also sources of iron. Only buckwheat and brown rice noodles were sources of niacin and thiamin. No pasta/noodles were a source of folate. The plain GF pizza crust was similar in macronutrient and vitamin content to GF buns and other breads but was not a source of minerals. The nutrient contributions of toppings to commercially available GF pizza reflected the addition of cheese, meats, and vegetables, as expected. GF pizza was a high source of fibre and an excellent source of calcium, vitamin B_12_, folate, and thiamin (Tables S1 and S2). Commercial pizza crust ingredients included diverse ingredients such as xanthan gum, rice and brown rice flours, millet flour, starches, chickpea flour, flax, psyllium, and pea protein, which likely contributed to the fibre and micronutrient contents.

### 3.4. Sweets and Snacks

Table 3 reports the energy and macronutrient composition of GF sweets and snack products. Energy, fat, cholesterol, and total sugar contents in this category tended to be higher than other staple GF grain categories (Table 1 and Table 2). Crackers were low in total saturated fat cholesterol but were not a source of fibre or micronutrients. Micronutrient content varied considerably between sub-categories of sweets and snacks (Tables S1 and S2). For example, energy bars were relatively micronutrient dense in comparison to baked goods (e.g., cakes, cookies, muffins). Muffins and energy/granola bars were the only products enriched with folic acid in this category. Energy bar ingredients such as nuts, seeds, flax, crisp brown rice, dates, raisins, and oats also contributed to the nutrient density of these products. Some cakes and muffins were sources of fibre, iron, magnesium, phosphorus, zinc, vitamin B_12_, niacin, riboflavin, and thiamin. This is likely explained by the addition of ingredients such as brown rice flour, amaranth flour, quinoa flour, xanthan gum, chickpea flour, psyllium, and almond flour to these products. Pretzels, crackers, and pancake mix were particularly high in sodium content (>600 mg Na/100 g), while most other products ranged from 200 to 400 mg Na/100 g.

## 4. Discussion

This work was focused on developing a comprehensive nutrient database by evaluating the product ingredients as well as the composition of commercially available GF products in the Canadian marketplace. Across all twelve categories evaluated, protein contents were consistently low, with the lowest values registered for cooked pasta or noodles, 2.4 ± 0.7 g/100 g, whereas the highest values were registered for non-fortified granola samples, 10.9 ± 2.9 g/100 g (Table 1). The low protein content in cooked pasta may be attributed to the chemical composition of the samples. Glutenin and gliadin are the two important protein fractions found in gluten, and they form a unique network when gluten-containing products are hydrated [17]. However, Phongthai et al. (2017) proposed that the absence of gluten in pasta results in a looser structure allowing for the faster penetration of water and swelling of starch and resulting in more cooking loss. The authors further explain that because water is used as a medium for cooking, some soluble proteins and soluble dietary fibre may also be lost during cooking [18]. This report may also provide support for the lower protein (2.4 ± 0.7 g vs. 7.9 ± 2.4 g) and fiber contents (3.6 ± 2.5 g vs. 1.1 ± 0.8 g) observed in the cooked versus the dry pasta, respectively (Table 2). Low protein contents in GF products have also been reported in similar studies in Spain [19,20]. In a recent study by Fabek et al. (2021), 51.2% of Canadians (representing the national average) reported that meat (51.9%), dairy (17.5%), and fish (7.1%) are among the top three sources of proteins that they consumed [21]. These findings may be important to limit concerns about the low protein contents of the cereal and grain-based GF products evaluated in this study since individuals with celiac disease can safely consume meat and dairy products as long as they are prepared without gluten-containing ingredients [6].

Changes to the new Canada’s Food Guide emphasize increased servings of plant-based proteins including pulses, seeds, and nuts in the diet [22,23]. These new guidelines may present an opportunity for food product developers in Canada to consider substituting traditional carbohydrate-rich flours (rice and potato) with more protein-rich alternatives (chickpea and quinoa) in reformulated GF baked products such as bagels and cookies to improve their nutritional profile [17]. The use of protein-rich flours could also improve the amino acid profiles of these products since gluten is deficient in the essential amino acid lysine and cannot be considered a complete protein source [24,25]. These new product formulations could also incorporate ingredients such as emulsifiers (e.g., lecithin), hydrocolloids (gums), and enzymes that may be necessary to induce network formation between polymers, which, in turn, would improve the volume, crumb texture, and overall sensory attributes of these foods [24]. In the baked products evaluated, xanthan gum and guar gum were already being incorporated in the formulations as thickening and gelling agents.

As expected, the carbohydrate contents were high (45.2 to 77.9%) in all the food categories evaluated, and the average energy values ranged from 109 ± 3.8 to 456 ± 32.6 kCal per 100 g. Examples of energy-dense foods evaluated include dried pastas and sweets, as well as energy bars, which registered average energy values of 425 ± 42.6 kCal per 100 g. In the diet, the primary role of carbohydrates (sugars and starches) is to provide energy to the cells in the body. However, in a food matrix, these ingredients have different functions. Starch is the most important cereal polysaccharide and is formed by two glucose polymers, amylose, and amylopectin, which is important for its thickening properties [26]. During the baking of GF products, starch undergoes gelatinization and pasting, which improves the structure of the products. Sugars are also involved in Maillard browning reactions that provide colors and aromas, which improve the sensory quality of GF products [25]. In GF breads, sugar acts as a source of nutrients for yeasts, which facilitates leavening, and in cakes and cookies, it binds with water and locks in moisture and contributes to a softer texture [27].

In spite of their important functional roles in establishing the sensory qualities of GF products, some authors have questioned whether GF products are healthier than non-GF foods given their high content of sugars, saturated fats, and sodium [28]. Indeed, this question has been the focus of several studies in different countries. For example, Lavriša et al. (2020) compared the nutrient content of GF and non-GF foods using a dataset that contained information on prepared foods in the Slovenian food supply [29]. Using a different study design, Babio et al. (2020) recorded 216 brands of GF products in Spain and compared their nutritional composition and cost to non-GF foods [30]. Both studies reported lower protein contents in the GF products; however, Babio et al. (2020) reported higher amounts of fats and sugar in some GF products. Similar to the Lavriša et al. (2020) study, our own observations registered low protein and sugar contents in GF products. Sweetened cereals, cakes, brownies, quick breads, and cookies had the highest total sugar contents (Table 2).

With regard to the fat content, the highest amounts were observed in crackers, granola bars, cakes, and muffins (Table 2). The main sources of fat in the cakes were whole eggs and vegetable oil (high monounsaturated canola oil or palm/soy shortening as well as palm oil) for the chocolate chip cookies. Oleic sunflower oil (>70%) was also observed in Pretzel chips, whereas organic coconut oil was observed in GF crackers. Sources of fat in bread products included seeds (sunflower and flax), eggs, and high monounsaturated canola oil. Fats play an important role in improving the flavor, texture, and appearance of foods. However, the choice of fat selected is important and can have negative health consequences. Calvo-Lerma et al. (2019) raised concerns over the use of oxidized palm oil (derived from heating the oil), which has been associated with cytotoxic damage and an increased risk of cancer [19]. Canadian recommendations for fat intake for adults 19 years of age and older is 20 to 35% of the daily energy intake, and high intakes of saturated fats have been strongly associated with cardiovascular disease [31]. Thus, it is desirable to keep saturated fatty acid intakes below the recommended values. The low saturated fat contents in the data presented further emphasizes the importance of optimizing formulations to ensure the ingredients selected provide the best functional and nutritional properties.

Other nutritional concerns that have been raised in the literature are related to the fibre content of GF foods, and are linked to the perceived replacement of wheat with refined starches as well as observational studies of low fibre intakes on GF diets [6,32]. Most of the GF breads and cereals in this study were a source (or high source) of fibre, regardless of being labelled as a white or whole grain product, because of the addition of functional food ingredients (e.g., psyllium husk and chicory root). Similar findings were reported by the Italian GF food composition database [9]. Among pastas, only cooked brown rice and buckwheat noodles were at least a source of fibre, while pasta blends made from corn, rice, and other gluten-free grain flours did not meet the fibre content claim. In addition, chocolate cake and blueberry muffins were a source of fibre due to the use of whole grain GF flour ingredients, psyllium, nut, and legume flours in these products. Thus, it appears that the inclusion of more nutritious GF ingredients (instead of refined starches) may be contributing to the nutrient density of select commercial GF products in Canada.

Among the micronutrients evaluated, the B vitamin (particularly folate) content of the GF diet has been a concern [6] due to the exclusion of enriched wheat flour and the non-mandatory enrichment of GF alternatives in Canada and other countries [25,32]. In the products evaluated here, product enrichment appeared sporadic and arbitrary in terms of micronutrients selected, especially among breads and cereals. In contrast, nutrition content claims of sources of vitamin B_6_, thiamin, riboflavin, and niacin were similar between enriched and unenriched sub-categories, likely due to the choice of functional fibre, egg, and/or whole grain ingredients in breads and cereals. Zinc, magnesium, and phosphorus content claims also reflected the inclusion of whole grain and other nutrient-dense ingredients. Overall, most GF product categories were categorized as a source of thiamin and riboflavin, and approximately half were a source of niacin, iron, or magnesium, but few were a source of folate, zinc, or calcium. Taken together, these findings suggest that the selection of enriched or fortified GF foods and a thorough review of the ingredient list for nutrient-dense ingredients may be useful strategies to improve nutrient intakes for those requiring a GF diet. However, special attention should be paid to folate adequacy given the high concern for inadequacy and relatively low content in GF grain products. Enriching the micronutrient content of GF products is another opportunity for food product developers to add value to these foods.

Although the estimation method used in this study is less precise than the direct chemical analysis of foods, the present study has several strengths. First, a rigorous and systematic approach was taken to evaluate whole food ingredient data obtained from a nationally representative food composition database to provide a comprehensive set of nutrient intakes. Appropriate nutrient retention factors were applied to all products to account for nutrient losses during processing. For composite foods, cooking yields were applied at the recipe level and nutrient retention adjusted per ingredient, as appropriate. Values for cooked pasta were extrapolated from dried pasta NFP using both cooking yield and nutrient retention adjustments. Sub-category averages are provided to facilitate dietary analysis when specific brands or characteristics of foods are unknown. Finally, food composition data are provided ‘as prepared’ in addition to dry weight ‘as sold’ to facilitate use of the tool in dietetic practice.

Nevertheless, there are some important limitations that should be noted. For example, specific brands and products used in the database may not represent all products available across the country (e.g., local health food stores and local markets) and product formulations may change over time. Indeed, some GF sub-categories could not be included due to the lack of sample size availability. Thus, specific, or novel GF foods may not be available within the database. Most importantly, estimated nutrient values are not directly comparable to chemically analyzed foods. Therefore, caution should be employed when comparing nutrient intake estimations from the database to direct nutrient data. Future research should directly chemically analyze nationally representative GF foods and seek to enhance nutrient density in GF grain products.

## 5. Conclusions

This study has produced the first food composition database of GF products in Canada using a systematic estimation approach based on the ingredient list of commercial key foods. Comprehensive data on average energy, macronutrient, and micronutrient data for 33 types of GF grain-based foods are provided (see Supplemental Materials: Database spreadsheet). This addresses a key gap in knowledge, particularly around micronutrient values, for individual and population level GF dietary assessment until more data from direct chemical analysis of GF foods can be obtained. Investigations of Canadian GF diet quality and dietary patterns will no longer be limited to the core nutrients provided on the NFP. This tool will also be of interest to dietitians working with individuals requiring a GF diet as a therapeutic treatment. The GF diet is a necessary therapeutic intervention in a growing number of diseases, disorders, and conditions worldwide. The availability of reliable food composition data is foundational to accurate and reliable dietary assessment at the individual and population level. Understanding dietary adequacy and diet quality in these populations has been hindered by the lack of country-specific GF food composition data available. Thus, this database is a key tool to enhance GF diet assessment applications in Canada.

## Figures and Tables

**Table 1:** Energy and macronutrient content of gluten-free bread and cereal products per 100 g *.

Food	Energy (kCal)	Energy (kJ)	Water (g)	Carbohydrate (g)	Total Sugar (g)	Fibre (g)	Protein (g)	Total Fat (g)	Saturated Fat (g)	Cholesterol (mg)
Breads										
Enriched whole/multi-grain (*n* = 4)	262 (7.7)	1096 (32.2)	26.4 (11.3)	46.5 (6.6)	2.5 (0.9)	7.4 ^a^ (3.5)	4.8 (2.5)	7.2 (3.0)	1.1 ^c^ (0.7)	0 ^d^ (0)
Enriched white (*n* = 3)	253 (29.3)	1059 (123)	10.5 (1.6)	49.9 (3.5)	7.0 (7.8)	7.2 ^a^ (3.5)	4.6 (1.4)	5.2 (1.0)	1.4 ^c^ (0.8)	0 ^d^ (0)
Unenriched, whole/multi-grain (*n* = 6)	249 (41.1)	1042 (172)	35.7 (5.4)	42.2 (5.4)	4.7 (3.9)	7.0 ^a^ (4.8)	4.8 (1.5)	7.2 (4.2)	0.9 ^c^ (1.0)	0 ^d^ (0)
Unenriched, white (*n* = 6)	266 (31.1)	1113 (130)	36.8 (6.0)	46.9 (7.3)	5.8 (3.6)	6.5 ^a^ (4.1)	3.9 (1.6)	8.1 (4.6)	1.2 ^c^ (1.5)	0 ^d^ (0)
Average (*n* = 19)	258 (30.0)	1079 (126)	30.1 (11.5)	45.9 (6.2)	4.9 (4.1)	7.0 ^a^ (3.8)	4.5 (1.7)	7.2 (3.6)	1.1 ^c^ (1.1)	0 ^d^ (0)
Bagels										
Unenriched, white (*n* = 4)	268 (46.4)	1121 (194)	30.8 (3.9)	47.2 (4.5)	4.5 (1.7)	3.5 ^b^ (1.2)	5.0 (3.2)	6.7 (2.8)	0.5 ^c^ (0.2)	0 ^d^ (0)
Unenriched, whole/multi-grain (*n* = 3)	231 (53.7)	967 (225)	35.2 (3.4)	42.1 (3.6)	4.4 (2.1)	3.7 ^b^ (1.5)	5.4 (1.6)	6.4 (3.6)	0.6 ^c^ (0.4)	0 ^d^ (0)
Average (*n* = 7)	252 (49.4)	1054 (207)	32.7 (4.2)	45.2 (4.7)	4.5 (1.7)	3.6 ^b^ (1.2)	5.2 (2.4)	6.6 (2.9)	0.5 ^c^ (0.3)	0 ^d^ (0)
English Muffins										
Unenriched (*n* = 4)	273 (31.1)	1142 (130)	31.3 (3.9)	53.2 (7.1)	3.6 (4.6)	1.8 (1.2)	4.3 (1.7)	3.9 (4.2)	0.3 ^c^ (0.2)	0 ^d^ (0)
Enriched (*n* = 3)	243 (16.2)	1017 (67.8)	34.0 (3.9)	46.3 (3.8)	7.6 (2.0)	1.2 (1.3)	5.2 (1.0)	3.9 (1.9)	0.4 ^c^ (0.1)	0.3 ^d^ (0.6)
Average (*n* = 7)	260 (28.6)	1088 (120)	32.5 (3.9)	50.2 (6.6)	5.3 (4.0)	1.5 (1.2)	4.7 (1.4)	3.9 (3.2)	0.3 ^c^ (0.2)	0.1 ^d^ (0.4)
Hamburger/hotdog Buns										
Unenriched (*n* = 5)	256 (30.3)	1071 (127)	32.7 (6.3)	47.2 (10.0)	8.4 (1.6)	5.5 ^a^ (1.3)	3.7 (2.1)	6.9 (1.3)	0.6 ^c^ (0.2)	0 ^d^ (0)
Enriched (*n* = 6)	244 (12.6)	1021 (52.7)	28.9 (12.9)	52.7 (2.2)	3.9 (1.9)	6.1 ^a^ (1.8)	2.8 (1.1)	3.5 (0.7)	0.5 ^c^ (0.2)	0 ^d^ (0)
Average (*n* = 11)	250 (22.0)	1046 (92.0)	30.6 (10.1)	50.2 (7.1)	5.9 (2.9)	5.8 ^a^ (1.6)	3.2 (1.6)	5.0 (2.0)	0.5 ^c^ (0.2)	0 ^d^ (0)
Breakfast Cereals										
Unsweetened, fortified (*n* = 5)	373 (16.7)	1561 (69.9)	4.3 (4.7)	83.1 (5.1)	8.7 (6.6)	6.7 ^b^ (6.0)	8.7 (3.7)	3.3 (2.6)	0.9 ^c^ (0.8)	0 ^d^ (0)
Sweetened, fortified (*n* = 5)	400 (15.3)	1674 (64.0)	5.2 (2.7)	82.1 (2.8)	27.9 (8.3)	5.4 (3.1)	6.7 (1.4)	5.3 (2.8)	0.7 ^c^ (0.3)	0 ^d^ (0)
Unsweetened, non-fortified (*n* = 7)	390 (33.4)	1632 (140)	6.5 (4.3)	82.0 (3.1)	12.8 (8.0)	6.3 (3.4)	7.9 (2.1)	3.6 (1.4)	0.7 ^c^ (0.6)	0 ^d^ (0)
Average (*n* = 17)	388 (25.7)	1623 (207.5)	5.5 (3.9)	82.3 (3.5)	16.1 (10.8)	6.2 (4.0)	7.8 (2.5)	4.0 (2.3)	0.8 ^c^ (0.6)	0 ^d^ (0)
Granola, non-fortified (*n* = 5)	456 (32.6)	1908 (136)	13.9 (18.1)	64.6 (5.8)	18.5 (1.7)	6.9 ^b^ (1.7)	10.0 (2.9)	18.4 (6.0)	2.7 ^c^ (1.2)	0 ^d^ (0)

* Data represent mean (standard deviation). ^a^ High source of fibre (>4 g per Health Canada reference serving). ^b^ Source of fibre (>2 g per Health Canada reference serving). ^c^ Low in saturated fat (<2 g per Health Canada reference serving). ^d^ Free of cholesterol (<2 mg per Health Canada reference serving).

**Table 2:** Energy and macronutrient content of gluten-free pastas and pizzas per 100 g *.

Food	Energy (kCal)	Energy (kJ)	Water (g)	Carbohydrate (g)	Total Sugar (g)	Fibre (g)	Protein (g)	Total Fat (g)	Saturated Fat (g)	Cholesterol (mg)
Pasta or Noodles, dried										
Brown rice/brown rice noodle (*n* = 5)	362 (8.4)	1515 (35.1)	11.8 (1.1)	78.8 (2.9)	0.3 (0.2)	3.4 (1.4)	7.0 (1.1)	2.8 ^c^ (1.1)	0.6 ^d^ (0.4)	0 ^e^ (0)
Ancient or mixed grain noodles (*n* = 5)	359 (3.3)	1502 (13.8)	10.9 (0.4)	78.1 (0.7)	0.4 (0.3)	2.3 (0.8)	7.5 (1.5)	1.9 ^c^ (0.3)	0.4 ^d^ (0.1)	0 ^e^ (0)
White rice/corn pasta noodles (*n* = 5)	358 (12.7)	1496(53.1)	12.0 (1.9)	80.8 (2.5)	0.3 (0.5)	2.3 (1.0)	6.5 (0.7)	1.2 ^c^ (0.6)	0.3 ^d^ (0.1)	0 ^e^ (0)
Buckwheat noodles (*n* = 3)	344 (18.5)	1439 (77.4)	12.6 (2.6)	71.4 (9.9)	2.5 (1.0)	8.2 ^b^ (2.4)	12.5 (1.9)	2.2 ^c^ (0.7)	0.5 ^d^ (0.1)	0 ^e^ (0)
Average (*n* = 18)	357 (11.7)	1493 (49.0)	11.7 (1.5)	77.9 (5.0)	0.7 (0.9)	3.6 (2.5)	7.9 (2.4)	2.0 ^c^ (0.9)	0.4 ^d^ (0.2)	0 ^e^ (0)
Pasta or Noodles, cooked										
Brown rice/brown rice noodle (*n* = 5)	110 (2.6)	460 (10.9)	73.1 (0.3)	24.1 (0.9)	<0.1 (<0.1)	1.1 ^b^ (0.4)	2.1 (0.3)	0.8 ^c^ (0.3)	0.2 ^d^ (0.1)	0 ^e^ (0)
Ancient or mixed grain noodles (*n* = 5)	111 (1.9)	464 (7.9)	72.9 (0.1)	24.1 (0.4)	0.1 (<0.1)	0.7 (0.2)	2.3 (0.4)	0.6 ^c^ (<0.1)	0.1 ^d^ (<0.1)	0 ^e^ (0)
White rice/or corn pasta noodles (*n* = 5)	109 (3.9)	456 (16.2)	73.2 (0.6)	24.7 (0.8)	0.1 (0.1)	0.7 (0.3)	2.0 (0.2)	0.4 ^c^ (0.2)	<0.1 ^d^ (<0.1)	0 ^e^ (0)
Buckwheat noodles (*n* = 3)	105 (5.6)	439 (23.4)	73.3 (0.8)	21.8 (3.0)	0.8 (0.3)	2.5 ^a^ (0.7)	3.8 (0.6)	0.7 ^c^ (0.2)	0.1 ^d^ (<0.1)	0 ^e^ (0)
Average (*n* = 18)	109 (3.8)	456 (15.8)	73.1 (0.5)	23.9 (1.6)	0.2 (0.3)	1.1 ^b^ (0.8)	2.4 (0.7)	0.6 ^c^ (0.3)	0.1 ^d^ (<0.1)	0 ^e^ (0)
Pizza Crust										
GF pizza crust, commercial (*n* = 5)	243 (73.9)	1017 (309)	39.4 (15.6)	44.1 (11.7)	3.4 (1.4)	2.7 ^a^ (1.2)	4.3 (1.5)	4.8 (3.3)	1.5 ^d^ (1.8)	27 (26)
Commercial Prepared Pizza with Toppings										
Frozen pizza, various toppings (*n* = 6)	246 (17.1)	1029 (71.5)	43.6 (5.5)	28.8 (6.3)	3.5 (2.0)	2.0 ^a^ (0.9)	8.5 (3.7)	10.5 (1.9)	4.8 (1.7)	17 (16)

* Data represent mean (standard deviation). ^a^ High source of fibre (>4 g per Health Canada reference serving). ^b^ Source of fibre (>2 g per Health Canada reference serving). ^c^ Low in fat (<3 g per 100g and <30% energy as fat). ^d^ Low in saturated fat (<2 g per Health Canada reference serving). ^e^ Free of cholesterol (<2 mg per Health Canada reference serving).

**Table 3:** Energy and macronutrient content of gluten-free sweets and snacks per 100 g *.

Food	Energy (kCal)	Energy (kJ)	Water (g)	Carbohydrate (g)	Total Sugar (g)	Fibre (g)	Protein (g)	Total Fat (g)	Saturated Fat (g)	Cholesterol (mg)
Crackers										
Crackers (*n* = 8)	438 (46.9)	1833 (196)	6.8 (4.0)	71.0 (11.9)	3.0 (2.7)	6.1 (4.6)	7.4 (3.3)	15.0 (7.1)	4.8 ^d^ (4.4)	4.2 ^e^ (7.7)
Rice cake/cracker (*n* = 5)	432 (41.5)	1807 (174)	9.1 (2.2)	79.2 (6.8)	0.3 ^a^ (0.4)	1.4 (1.9)	9.1 (3.3)	7.3 (8.1)	0.5 ^d^ (0.7)	0 ^e^ (0)
Pretzels (*n* = 5)	396 (25.6)	1657 (107)	3.5 (1.0)	77.0 (3.7)	2.4 ^a^ (2.3)	2.7 (2.6)	3.2 (2.1)	8.8 (2.3)	2.5 ^d^ (2.0)	0 ^e^ (0)
Average (*n* = 18)	425 (42.6)	1778 (178)	6.5 (3.5)	74.9 (9.3)	2.1 ^a^ (2.4)	3.9 (4.0)	6.7 (3.7)	11.1 (7.1)	3.0 ^d^ (3.5)	1.9 ^e^ (5.4)
Energy or Granola Bars										
Energy/granola bars (*n* = 5)	428 (49.4)	1791 (207)	8.0 (1.9)	54.7 (7.6)	32.6 (7.1)	8.0 ^b^ (2.1)	14.3 (6.7)	17.9 (8.7)	3.2 ^d^ (1.5)	0 ^e^ (0)
Pancakes and Waffles										
GF pancake mix (*n* = 6)	349 (14.8)	1460 (61.9)	6.8 (2.7)	79.5 (3.7)	9.6 (6.6)	2.7 (1.7)	4.3 (1.5)	1.1 ^c^ (1.0)	0.3 ^d^ (0.3)	0 ^e^ (0)
GF pancakes, prepared from mix ^#^ (*n* = 6)	229 (17.5)	958 (73.2)	46.7 (4.0)	39.5 (3.5)	5.5 (2.5)	1.3 (0.9)	5.8 (2.0)	5.0 (1.7)	2.1 ^d^ (1.2)	103 (62)
GF waffles, frozen or as prepared from mix * (*n* = 5)	251 (57.8)	1050 (242)	38.3 (15.2)	42.0 (9.5)	5.0 (1.7)	1.0 (0.6)	4.2 (2.6)	6.7 (3.7)	1.6 ^d^ (0.5)	74 (69)
Quick Bread, Cakes, and Cookies										
Chocolate cake/cupcake (*n* = 5)	339 (69.4)	1418 (290)	25.0 (5.7)	47.6 (5.9)	31.8 (5.5)	2.7 ^b^ (0.8)	4.9 (0.5)	15.2 (7.3)	4.7 (3.6)	53 (38)
White cake/cupcake (*n* = 5)	333 (24.9)	1393 (104)	26.9 (4.7)	52.0 (8.1)	29.4 (3.1)	1.0 (0.8)	3.4 (0.8)	12.5 (1.9)	3.9 (2.3)	78 (16)
Brownies, commercial or as prepared from mix ^#^ (*n* = 5)	414 (53.4)	1732 (223)	14.7 (6.0)	59.0 (3.3)	41.7 (3.0)	3.3 (2.2)	4.3 (0.7)	18.4 (7.7)	5.2 (1.8)	57 (34)
Muffin, blueberry (*n* = 5)	348 (42.2)	1456 (177)	24.7 (11.9)	52.4 (18.9)	23.8 (9.3)	2.1 ^b^ (1.8)	5.4 (1.9)	13.4 (8.7)	2.1 (1.2)	53 (35)
Banana bread (*n* = 4)	321 (90.4)	1343 (378)	33.8 (13.7)	45.1 (4.5)	22.5 (6.8)	1.8 (0.4)	4.1 (1.7)	14.0 (7.3)	2.9 ^d^ (1.9)	52 (10)
Cookies, chocolate chip (*n* = 5)	206 (163)	862 (682)	23.8 (14.2)	24.8 (28.7)	14.7 (11.1)	1.1 (1.0)	2.9 (1.7)	10.6 (4.8)	2.4 ^d^ (0.7)	25 (29)

* Data represent mean (standard deviation). ^#^ As prepared according to package instructions to add egg, vegetable oil or butter, and milk or water. ^a^ Free of sugars (<0.5 g per Health Canada reference serving). ^b^ Source of fibre (>2 g per Health Canada reference serving). ^c^ Low in fat (<3 g per 100g and <30% energy as fat). ^d^ Low in saturated fat (<2 g per Health Canada reference serving). ^e^ Free of cholesterol (<2 mg per Health Canada reference serving).

## Data Availability

The data presented in this study are available as online supplemental material.

## Supplementary Materials

The following supporting information can be downloaded at: https://www.mdpi.com/article/10.3390/foods11152215/s1, Table S1: Lipid and mineral content of commercial gluten-free grain products per 100 g; Table S2: Vitamin content of commercial gluten-free grain products per 100 g; Database spreadsheet: Canadian gluten-free food composition database; Raw data file: GF database raw data file.
