# Supplementary material for: Development of a Canadian Food Composition Database of Gluten-Free Products
Source: Foods. 2022 Jul 26;11(15):2215. doi: 10.3390/foods11152215 (PMC9332361; doi:10.3390/foods11152215)
Supplement: Supplementary file 1 [file foods-11-02215-s001.zip › Foods-S1809780 Supplemental Tables S1-S2.pdf]

foods MDPI

Supplementary Materials: The following supporting information can be downloaded at: https://www.mdpi.com/article/10.3390/foods11152215/s1,

Table S1: Lipid and mineral content of commercial gluten-free grain products per 100 grams

Table S2: Vitamin content of commercial gluten-free grain products per 100 grams

Table S1. Lipid and mineral content of commercial gluten-free grain products per 100 grams*

**Food**	**MUFA (g)**	**PUFA (g)**	**PFA 18:2 (g)**	**PFA 18:3 (g)**	**Calcium (mg)**	**Iron (mg)**	**Magnesium (Mg)**	**Phosphorus (mg)**	**Potassium (mg)**	**Sodium (mg)**	**Zinc (mg)**
Breads											
Enriched whole/multi-grain (n=4)	2.8 (1.1)	3.0 (1.2)	1.9 (1.0)	1.1 (0.4)	94.4[a] (34.3)	2.6[a] (0.9)	53.5[a] (28.9)	113.1[a] (36.0)	97.5 (74.5)	434 (105)	0.9a (0.3)
Enriched white (n=3)	1.5 (0.6)	2.3 (1.3)	2.0 (1.1)	0.4 (0.1)	132.4[a] (13.3)	2.7[a] (0.5)	26.0 (10.6)	60.4 (4.0)	96.0 (102.9)	425 (60)	0.5 (<0.1)
Unenriched, whole/multi-grain (n=6)	3.4 (1.9)	1.9 (0.9)	1.3 (0.6)	0.8 (0.5)	53.4 (33.5)	1.3[a] (0.7)	39.1[a] (18.7)	96.0[a] (42.8)	115.3 (23.5)	440 (95)	0.8a (0.4)
Unenriched, white (n=6)	4.3 (2.5)	1.7 (1.1)	1.4 (0.8)	0.3 (0.5)	22.1 (26.0)	1.4[a] (0.9)	16.7 (5.3)	49.9 (13.4)	72.0 (23.9)	482 (122)	0.5 (0.1)
Average (n=19)	3.2 (2.0)	2.1 (1.1)	1.6 (0.8)	0.6 (0.5)	64.6 (48.2)	1.8[a] (1.0)	33.0[a] (21.4)	79.4 (38.3)	94.8 (52.2)	450 (97)	0.7 (0.3)
Bagels											
Unenriched, white (n=4)	3.6 (1.9)	1.9 (0.8)	1.5 (0.6)	0.3 (0.4)	33.8 (44.5)	0.7 (<0.1)	45.1[a] (32.6)	148.0[a] (71.2)	112.1 (42.4)	517 (69)	0.8[a] (0.3)
Unenriched, whole/multi-grain (n=3)	3.1 (2.2)	1.9 (1.0)	1.3 (0.7)	0.4 (0.7)	52.0 (38.3)	1.1[a] (0.6)	36.6[a] (16.6)	80.3[a] (23.0)	198.2 (49.6)	482 (126)	0.6 (0.2)
Average (n=7)	3.3 (1.9)	1.9 (0.9)	1.4 (0.6)	0.4 (0.5)	41.6 (39.6)	0.9 (0.4)	41.5[a] (25.4)	118.9[a] (63.4)	149.0 (61.9)	502 (90)	0.7[a] (0.3)
English Muffins											
Unenriched (n=4)	1.9 (2.2)	1.5 (1.4)	0.9 (0.7)	0.5 (0.7)	67.0 (46.5)	1.3 (0.4)	43.4 (16.5)	156.9 (17.8)	152.5 (24.5)	433 (155)	1.3[a] (0.7)
Enriched (n=3)	1.8 (0.7)	1.4 (1.1)	0.9 (0.6)	0.5 (0.5)	128.6 (119.7)	1.3 (0.3)	26.2 (17.5)	123.7 (78.5)	163.6 (100.6)	582 (181)	0.7 (0.3)
Average (n=7)	1.9 (1.6)	1.5 (1.2)	0.9 (0.6)	0.5 (0.6)	93.4 (83.3)	1.3 (0.3)	36.6 (18.2)	142.7 (50.3)	157.2 (60.9)	497 (171)	1.0[a] (0.6)
Hamburger/hotdog Buns											
Unenriched (n=5)	3.4 (0.8)	2.2 (0.7)	1.9 (0.8)	0.2 (0.3)	64.2 (61.1)	0.8 (0.5)	35.3[a] (11.6)	93.6[a] (20.5)	142.3 (93.0)	475 (23)	0.8[a] (0.2)
Enriched (n=6)	1.8 (0.4)	0.9 (0.1)	0.7 (<0.1)	0.2 (0.2)	162.7 (62.2)	4.0[a] (1.5)	17.2 (2.7)	56.9 (7.7)	55.9 (45.8)	488 (49)	0.5 (<0.1)
Average (n=11)	2.5 (1.0)	1.5 (0.8)	1.3 (0.8)	0.2 (0.2)	117.9 (77.9)	2.5 (2.0)	25.4 (12.1)	73.6 (23.8)	95.2 (80.9)	482 (38)	0.6 (0.2)

Food **MUFA (g)** **PUFA (g)** **PFA 18:2 (g)** **PFA 18:3 (g)** **Calcium (mg)** **Iron (mg)** **Magnesium (Mg)** **Phosphorus (mg)** **Potassium (mg)** **Sodium (mg)** **Zinc (mg)**

Breakfast Cereals											
Unsweetened, fortified (n=5)	0.9 (0.7)	1.0 (0.8)	1.0 (0.7)	<0.1 (<0.1)	152.5 (189.3)	17.1^{b} (3.8)	146.0^{a} (94.0)	273.6^{a} (119.6)	255.5 (99.1)	687 (102)	2.1^{a} (0.8)
Sweetened, fortified (n=5)	2.2 (1.6)	1.7 (0.6)	1.5 (0.4)	0.2 (0.2)	371.8^{a} (47.4)	16.8^{b}(2.2)	108.2^{a} (41.1)	221.8^{a} (106.7)	203.5 (72.5)	551 (139)	2.2^{a} (0.8)
Unsweetened, non-fortified (n=7)	0.9 (0.4)	1.4 (0.7)	1.1 (0.4)	0.3 (0.6)	15.2 (8.9)	3.0^{a} (1.2)	101.3^{a} (42.6)	221.8^{a} (92.3)	263.5 (79.0)	380^{d} (174)	1.8 (0.5)
Average (n=17)	1.3 (1.1)	1.4 (0.7)	1.2 (0.5)	0.2 (0.4)	160.4 (181.0)	11.2 (7.4)	116.5 (60.9)	237.0 (101.4)	243.5 (82.6)	520.8 (190.8)	2.0 (0.7)
Granola, non-fortified (n=5)	6.9 (3.3)	7.5 (2.7)	5.7 (2.1)	0.9 (0.8)	60.8 (28.2)	3.1^{a} (1.2)	114.3^{a} (31.9)	335.8^{a} (57.3)	318.0 (48.3)	101 (28)	2.4^{a} (0.6)
Pasta or Noodles, dried											
Brown rice/brown rice noodle (n=5)	0.8 (0.3)	0.8 (0.3)	0.8 (0.3)	<0.1 (<0.1)	24.4 (18.4)	1.3 (0.6)	126.3^{a} (58.3)	263.6^{a} (71.3)	223.6 (66.5)	2.3^{d} (1.6)	1.9 (0.6)
Ancient or mixed grain noodles (n=5)	0.5 (0.1)	0.7 (0.1)	0.7 (0.1)	<0.1 (<0.1)	20.0 (17.0)	2.6 (2.1)	85.1^{a} (7.4)	214.2 (27.2)	225.5 (70.2)	5.4^{d} (3.3)	1.6 (0.2)
White rice/corn pasta noodles (n=5)	0.3 (0.2)	0.5 (0.3)	0.5 (0.3)	<0.1 (<0.1)	4.5 (3.0)	0.8 (0.7)	45.9 (26.4)	137.0 (71.5)	120.2 (84.4)	2.7^{d} (3.6)	1.0 (0.4)
Buckwheat noodles (n=3)	0.7 (0.2)	0.7 (0.2)	0.7 (0.2)	<0.1 (<0.1)	44.8 (4.2)	3.1 (1.0)	246.7^{a} (7.2)	370.4^{a} (73.3)	507.5 (140.8)	8.1^{d} (2.5)	2.4^{a} (1.2)
Average (n=18)	0.6 (0.3)	0.7 (0.3)	0.6 (0.3)	<0.1 (<0.1)	21.1 (18.3)	1.8 (1.5)	112.6^{a} (75.8)	232.5 (98.1)	242.7 (152.3)	4.3^{d} (3.4)	1.6 (0.7)
Pasta or Noodles, cooked											
Brown rice/brown rice noodle (n=5)	0.3 (<0.1)	0.3 (<0.1)	0.2 (<0.1)	<0.1 (<0.1)	7.1 (5.3)	0.3 (0.1)	33.0^{b} (15.2)	80.4^{a} (21.8)	20.5 (6.1)	$<1.0^{d}$ (<1)	0.6^{a} (0.2)
Ancient or mixed grain noodles (n=5)	0.2 (<0.1)	0.2 (<0.1)	0.2 (<0.1)	<0.1 (<0.1)	5.8 (4.9)	0.6^{a} (0.5)	22.2^{a} (1.7)	66.3^{a} (9.2)	20.9 (6.4)	$<1.0^{d}$ (<1)	0.5^{a} (<0.1)
White rice/corn pasta noodles (n=5)	0.1 (<0.1)	0.1 (0.1)	0.1 (0.1)	<0.1 (<0.1)	1.3 (0.9)	0.2 (0.2)	12.0 (6.9)	41.8^{a} (21.8)	11.0 (7.7)	$<1.0^{d}$ (1.1)	0.3^{a} (0.1)
Buckwheat noodles (n=3)	0.2 (<0.1)	0.2 (<0.1)	0.2 (<0.1)	<0.1 (<0.1)	13.0 (1.2)	0.7^{a} (0.2)	64.4^{a} (1.9)	113.1^{b} (22.4)	46.5 (12.9)	1.2^{d} (0.4)	0.7^{a} (0.4)

Average (n=18)	0.2 (<0.1)	0.2 (<0.1)	0.2 (<0.1)	<0.1 (<0.1)	6.1 (5.3)	0.4[a] (0.3)	29.5[b] (19.8)	71.2[a] (30.0)	22.3 (13.9)	<1.0[d] (<1)	0.5[a] (0.2)
Pizza Crust											
GF pizza crust, commercial (n=5)	2.3 (1.4)	0.8 (0.4)	0.7 (0.3)	0.1 (0.2)	45.4 (45.9)	1.1 (0.9)	24.3 (10.6)	136.5 (129.1)	127.0 (53.4)	423 (186)	0.7 (0.2)
Commercial Prepared Pizza with Toppings											
Frozen pizza, various toppings (n=6)	4.6 (0.9)	1.9 (0.5)	1.2 (0.2)	0.8 (0.4)	198.0[c] (72.6)	1.1[a] (0.3)	29.5[a] (9.5)	140.0[a] (51.4)	185.1[a] (48.5)	486 (81)	1.0[b] (0.3)
**Food**	**MUFA (g)**	**PUFA (g)**	**PFA 18:2 (g)**	**PFA 18:3 (g)**	**Calcium (mg)**	**Iron (mg)**	**Magnesium (Mg)**	**Phosphorus (mg)**	**Potassium (mg)**	**Sodium (mg)**	**Zinc (mg)**
Crackers											
Crackers (n=8)	4.3 (2.8)	4.0 (2.5)	2.8 (1.7)	0.7 (0.6)	82.4 (105.8)	2.6 (1.5)	67.2 (64.9)	192.2 (124)	215.6 (157)	621 (297)	1.3 (0.8)
Rice cake/cracker (n=5)	3.9 (5.1)	2.1 (2.6)	1.8 (1.8)	0.4 (0.7)	12.0 (21.7)	0.7 (0.6)	79.6 (44.5)	194.3 (98)	144.8 (52.9)	195 (184)	1.3 (0.5)
Pretzels (n=5)	4.3 (2.4)	1.0 (0.5)	1.0 (0.4)	<0.1 (<0.1)	40.4 (24.2)	0.6 (0.4)	20.9 (17.1)	47.8 (24.0)	81.2 (105)	1330 (308)	0.4 (0.3)
Average (n=18)	4.2 (3.3)	2.7 (2.4)	2.0 (1.6)	0.4 (0.6)	51.1 (76.2)	1.5 (1.4)	57.8 (53.4)	152.7 (115)	158.6 (129)	700 (511)	1.1 (0.7)
Energy or Granola Bars											
Energy/granola bars (n=5)	8.0 (4.5)	6.2 (3.5)	4.8 (3.5)	0.8 (0.9)	98.4 (54.1)	2.4[a] (0.8)	139.3[a] (29.4)	285.9[a] (86.0)	542.4 (132)	88 (58)	2.3[a] (0.9)
Pancakes and Waffles											
Pancake mix (n=6)	0.4 (0.3)	0.4 (0.3)	0.4 (0.3)	<0.1 (<0.1)	84.3 (73.5)	1.8 (2.2)	40.5 (33.8)	295.0 (309)	107.7 (117)	813 (256)	0.8 (0.6)
Pancakes, prepared from mix[#] (n=6)	1.8 (0.6)	0.7 (0.2)	0.6 (0.2)	<0.1 (<0.1)	71.5 (44.8)	1.3[a] (1.2)	24.2 (18.8)	194.6 (162)	106.6 (74.3)	440 (94)	0.8[a] (0.4)
Waffles, frozen/prepared from mix[#] (n=5)	2.5 (1.7)	2.1 (2.1)	1.9 (2.0)	0.3 (0.3)	31.4 (14.1)	1.5[a] (1.3)	26.4 (12.3)	162.0 (79.9)	120.3 (44.4)	471 (122)	0.7[a] (0.2)
**Quick Bread, Cakes and Cookies**											
Chocolate cake/cupcake (n=5)	6.9 (3.3)	3.3 (1.4)	2.3 (0.6)	0.5 (0.4)	49.0 (14.8)	3.0[a] (2.0)	40.9[a] (15.0)	220.4[a] (74.7)	176.2 (79.8)	325 (161)	0.7[a] (0.2)
White cake/cupcake (n=5)	5.5 (1.3)	2.5 (1.1)	2.1 (1.0)	0.4 (0.2)	58.7 (34.0)	0.5 (0.2)	10.4 (1.8)	214.6 (126)	69.0 (48.0)	404 (104)	0.4 (<0.1)

Brownies, commercial or as prepared from mix[#] (n=5)	8.9 (5.7)	3.8 (2.6)	2.8 (1.7)	1.0 (1.0)	21.5[a] (10.3)	4.2[a] (1.1)	46.3 (20.9)	102.3 (30.2)	149.4 (97.7)	217 (88)	0.8 (0.2)
Muffin, blueberry (n=5)	7.1 (4.8)	3.7 (2.5)	3.0 (2.3)	0.7 (0.4)	70.4 (31.7)	1.0[a] (0.5)	28.8[a] (22.6)	220.8[a] (127)	157.0 (201)	387 (226)	0.6[a] (0.2)
Banana bread (n=4)	7.2 (3.9)	3.5 (1.6)	2.6 (1.0)	0.9 (0.6)	72.1 (43.4)	1.1 (0.8)	24.0 (9.4)	204.2 (74.8)	127.2 (112)	292 (61)	0.5 (0.3)
Cookies, chocolate chip (n=5)	5.5 (2.4)	2.6 (1.3)	1.8 (1.1)	0.7 (0.2)	57.7 (20.3)	1.0 (0.2)	16.7 (10.4)	140 (91.5)	119.7 (10.6)	176 (163)	0.4 (0.2)

* Data represent mean (standard deviation). [#]As prepared according to package instructions to add egg, vegetable oil or butter and milk or water.
[a] Source of vitamin or mineral (>5 % DV per Health Canada reference serving). [b] High source of vitamin or mineral (>15 % DV per Health Canada reference serving).
[c] Excellent source of vitamin or mineral (>25 % DV per Health Canada reference serving). [d] Low in sodium (<140 mg per Health Canada reference serving)

Table S2. Vitamin content of commercial gluten-free grain products per 100 grams*

Food	Vitamin A µg)	Vitamin B6 (mg)	Vitamin B12 (µg)	Vitamin C (mg)	Vitamin D (µg)	Folate DFE (µg)	Folic Acid (µg)	Food Folate (µg)	Niacin (mg)	Riboflavin (mg)	Thiamin (mg)
Breads											
Enriched whole/multi-grain (n=4)	0.01 (0.01)	0.16[a] (0.03)	0.0 (0)	<0.1 (<0.1)	0.0 (0.0)	124.0[c] (54.5)	56.5 (28.0)	27.9 (6.9)	2.4[a] (0.8)	0.17[a] (0.04)	0.2[a] (<0.1)
Enriched white (n=3)	0.18 (0.32)	0.12[a] (0.04)	0.0 (0)	0.0 (<0.1)	0.0 (0.0)	125.7[c] (36.5)	59.1 (23.2)	22.1 (1.9)	2.7[a] (0.1)	0.21[a] (0.03)	0.2[a] (<0.1)
Unenriched, whole/multi-grain (n=6)	0.02 (0.04)	0.15[a] (0.05)	0.0 (0)	0.2 (0.3)	0.0 (0.0)	26.5 (9.6)	0.0 (0.0)	26.5 (9.6)	1.4[a] (0.5)	0.14[a] (0.09)	0.3[b] (0.1)
Unenriched, white (n=6)	0.02 (0.06)	0.12[a] (0.03)	0.0 (0)	1.1 (2.5)	0.0 (0.0)	20.2 (3.6)	0.0 (0.0)	20.2 (3.6)	1.0 (0.3)	0.12[a] (0.08)	0.2[a] (0.1)
Average (n=19)	0.04 (0.13)	0.14[a] (0.03)	0.0 (0)	0.4 (1.4)	0.0 (0.0)	60.7[a] (56.6)	21.2 (31.7)	24.1 (6.9)	1.7[a] (0.8)	0.15[a] (0.07)	0.3[a] (0.1)
Bagels											
Unenriched, white (n=4)	0.06 (0.08)	0.25[a] (0.16)	0.0 (0)	<0.1 (<0.1)	0.0 (0)	25.7[a] (12.8)	0.0 (0.0)	25.7 (12.8)	2.1[a] (1.3)	0.08[a] (0.03)	0.3[b] (<0.1)
Unenriched, whole/multi-grain (n=3)	0.19 (0.20)	0.11[a] (0.01)	0.0 (0)	0.1 (0.2)	0.03 (0.06)	35.5[a] (6.8)	0.0 (0.0)	35.5 (6.8)	1.1[a] (0.3)	0.11[a] (0.08)	0.2[a] (<0.1)
Average (n=7)	0.12 (0.15)	0.19[a] (0.14)	0.0 (0)	<0.1 (0.1)	0.01 (0.04)	29.9[a] (11.1)	0.0 (0.0)	29.9 (11.1)	1.6[a] (1.1)	0.09[a] (0.05)	0.2[b] (<0.1)
English Muffins											
Unenriched (n=4)	21.5 (25.3)	0.18[a] (0.08)	0.2 (0.2)	0.2 (0.2)	0.3 (0.6)	19.6 (5.4)	0.0 (0.0)	19.8 (5.0)	2.3[a] (1.8)	0.13[a] (0.06)	0.2[a] (0.1)
Enriched (n=3)	40.0 (34.7)	0.11 (0.05)	0.3[a] (0.2)	0.3 (0.4)	0.7 (0.6)	21.5 (9.8)	2.1 (3.7)	17.9 (4.7)	1.5[a] (1.5)	0.23[a] (0.08)	0.2[a] (0.1)
Average (n=7)	29.4 (28.6)	0.15 (0.07)	0.2 (0.2)	0.2 (0.3)	0.5 (0.6)	20.4 (6.9)	0.9 (2.4)	18.9 (4.8)	2.0[a] (1.6)	0.17[a] (0.08)	0.2[a] (0.1)

Hamburger/hotdog Buns											
Unenriched (n=5)	0.08 (0.09)	0.14^{a} (0.04)	0.0 (0)	<0.1 (<0.1)	0.4 (0.8)	34.1^{a} (13.4)	0.0 (0.0)	34.1 (13.4)	1.4^{a} (0.3)	0.08^{a} (0.04)	0.3^{b} (<0.1)
Enriched (n=6)	0.03 (0.05)	0.12^{a} (0.01)	0.0 (0)	0.1 (0.2)	0.0 (0.0)	57.3^{a} (64.7)	13.9 (33.9)	28.0 (9.1)	2.7^{a} (1.2)	0.29^{b} (0.20)	0.4^{c} (0.3)
Average (n=11)	0.05 (0.07)	0.13^{a} (0.02)	0.0 (0)	<0.1 (0.2)	0.2 (0.5)	46.7^{a} (48.1)	7.6 (25.1)	30.8 (11.1)	2.1^{a} (1.1)	0.20^{a} (0.18)	0.4^{b} (0.2)

Food	Vitamin A (µg)	Vitamin B6 (mg)	Vitamin B12 (µg)	Vitamin C (mg)	Vitamin D (µg)	Folate DFE (µg)	Folic Acid (µg)	Food Folate (µg)	Niacin (mg)	Riboflavin (mg)	Thiamin (mg)
Breakfast Cereals											
Unsweetened, fortified (n=5)	1.6 (3.6)	0.6^{a} (0.1)	0.0 (0)	0.0 (0.0)	2.5 (5.6)	109.6^{a} (7.3)	54.6 (5.3)	16.7 (11.4)	4.3^{a} (1.0)	0.61^{a} (1.2)	1.3^{c} (0.8)
Sweetened, fortified (n=5)	1.2 (2.5)	0.6^{a} (0.3)	0.0 (0)	<0.1 (<0.1)	0.0 (0.0)	143.5^{a} (45.1)	78.0 (29.0)	10.6 (6.5)	4.7^{a} (3.1)	0.07 (0.02)	0.5^{b} (0.8)
Unsweetened, non-fortified (n=7)	12.1 (23.3)	0.4^{a} (0.2)	0.0 (0)	0.2 (0.6)	0.0 (0.0)	13.5 (13.0)	0.0 (0.0)	13.5 (13.0)	3.8^{a} (1.3)	0.09 (0.03)	0.3^{a} (<0.1)
Average (n=17)	5.8 (15.4)	0.5^{a} (0.2)	0.0 (0)	0.1 (0.4)	0.7 (3.0)	80.0^{a} (63.7)	35.9 (38.9)	13.6 (10.6)	4.2^{a} (1.9)	0.23^{a} (0.6)	0.7^{b} (0.7)
Granola, non-fortified (n=5)	12.6 (17.3)	0.3^{a} (0.1)	0.0 (0)	0.3 (0.6)	0.0 (0.0)	29.3 (8.7)	0.0 (0.0)	29.3 (8.7)	2.6^{a} (1.7)	0.11 (0.03)	0.4^{b} (0.1)
Pasta or Noodles, dried											
Brown rice/brown rice noodle (n=5)	0.0 (0.0)	0.44^{a} (0.17)	0.0 (0)	0.0 (0)	0.0 (0)	11.0 (3.0)	0.0 (0)	11.4 (3.0)	4.1^{a} (1.4)	0.06 (0.02)	0.28^{a} (0.10)
Ancient or mixed grain noodles (n=5)	2.3 (2.5)	0.38^{a} (0.04)	0.0 (0)	0.0 (0)	0.0 (0)	11.8 (1.8)	0.0 (0)	11.8 (1.8)	1.5 (0.8)	0.08 (0.04)	0.18 (0.05)
White rice/corn pasta noodles (n=5)	3.3 (4.2)	0.22 (0.13)	0.0 (0)	0.0 (0)	0.0 (0)	13.1 (12.2)	0.0 (0)	13.9 (12.1)	1.5 (0.8)	0.05 (0.02)	0.12 (0.07)
Buckwheat noodles (n=3)	0.0 (0.0)	0.51^{a} (0.01)	0.0 (0)	0.0 (0)	0.0 (0)	37.2 (1.1)	0.0 (0)	37.2 (1.1)	5.4^{a} (0.2)	0.17 (<0.1)	0.37^{a} (0.01)
Average (n=18)	1.6 (2.8)	0.37^{a} (0.15)	0.0 (0)	0.0 (0)	0.0 (0)	16.2 (11.5)	0.0 (0)	16.4(11.4)	2.8 (1.8)	0.08 (0.05)	0.22 (0.11)
Pasta or Noodles, cooked											
Brown rice/brown rice noodle (n=5)	0.0 (0.0)	0.11^{a} (0.04)	0.0 (0)	0.0 (0)	0.0 (0)	2.4 (0.6)	0.0 (0)	2.4 (0.6)	0.8^{a} (0.3)	0.01 (<0.01)	0.05^{a} (0.02)
Ancient or mixed grain noodles (n=5)	0.7 (0.8)	0.09^{a} (0.01)	0.0 (0)	0.0 (0)	0.0 (0)	2.5 (0.4)	0.0 (0)	2.6 (0.4)	0.3 (0.2)	0.02 (0.01)	0.04^{a} (0.01)
White rice/corn pasta noodles (n=5)	1.0 (1.2)	0.05^{a} (0.03)	0.0 (0)	0.0 (0)	0.0 (0)	2.8 (2.6)	0.0 (0)	3.0 (2.6)	0.3 (0.2)	0.01 (0.01)	0.02 (0.01)

Food	Vitamin A (µg)	Vitamin B6 (mg)	Vitamin B12 (µg)	Vitamin C (mg)	Vitamin D (µg)	Folate DFE (µg)	Folic Acid (µg)	Food Folate (µg)	Niacin (mg)	Riboflavin (mg)	Thiamin (mg)
Buckwheat noodles (n=3)	0.0 (0.0)	0.13^{b} (0)	0.0 (0)	0.0 (0)	0.0 (0)	7.9 (0.2)	0.0 (0)	7.9 (0.2)	1.1^{a} (<0.1)	0.04 (<0.01)	0.07^{a} (0.01)
Average (n=18)	0.5 (0.8)	0.37^{a} (0.15)	0.0 (0)	0.0 (0)	0.0 (0)	3.5 (2.5)	0.0 (0)	3.5 (2.4)	0.6^{a} (0.4)	0.02^{a} (0.01)	0.04^{a} (0.02)
Pizza Crust											
GF pizza crust, commercial (n=5)	14.4 (13.0)	0.16^{a} (0.04)	0.2 (0.2)	5.3 (11.7)	0.3 (0.4)	44.5^{a} (25.3)	5.6 (12.5)	35 (12)	1.7^{a} (1.2)	0.13^{a} (0.08)	0.3^{a} (0.2)
Commercial Prepared Pizza with Toppings											
Frozen pizza, various toppings (n=6)	33.7^{a} (31.4)	0.11^{a} (0.06)	0.3^{c} (0.3)	1.9 (1.4)	0.04 (0.06)	34.3^{b} (17.7)	2.0 (4.8)	34 (18)	1.1^{a} (0.6)	0.12^{b} (0.07)	0.2^{c} (<0.1)
Crackers											
Crackers (n=8)	1.9 (3.6)	0.24 (0.13)	0.07 (0.14)	0.2 (0.2)	0.05 (0.10)	30.3 (20.7)	0.0 (0)	30.3 (20.7)	1.5 (1.4)	0.07 (0.04)	0.2 (0.2)
Rice cake/cracker (n=5)	0.0 (0.0)	0.40 (0.06)	0.0 (0)	0.2 (0.4)	0.0 (0.0)	13.6 (10.7)	0.0 (0)	13.6 (10.7)	2.1 (1.4)	0.10 (0.04)	0.2 (0.1)
Pretzels (n=5)	0.7 (1.1)	0.12 (0.08)	0.0 (0)	7.8 (10.2)	0.0 (0.0)	22.3 (23.6)	0.0 (0)	22.1 (23.8)	0.5 (0.6)	0.04 (0.03)	0.1 (<0.1)
Average (n=18)	1.0 (2.5)	0.20 (0.14)	0.03 (0.09)	2.3 (6.1)	0.02 (0.07)	23.4 (19.6)	0.0 (0)	23.4 (19.7)	1.4 (1.5)	0.06 (0.04)	0.2 (0.1)
Energy or Granola Bars											
Energy/granola bars (n=5)	48.3 (106.2)	0.3^{a} (0.1)	0.2^{a} (0.5)	3.4 (4.3)	0.0 (0.0)	64.4 (40.3)	26.6 (36.5)	29.3 (17.2)	4.4^{a} (1.7)	0.3^{a} (0.2)	0.3^{a} (0.1)
Pancakes and Waffles											
Pancake mix (n=6)	0.03 (0.08)	0.25 (0.13)	<0.1 (<0.1)	3.8 (9.2)	0.1 (0.2)	13.9 (16.8)	0.0 (0.0)	13.9 (16.8)	1.0 (1.6)	0.05 (0.05)	0.12 (0.08)
Pancakes, prepared from mix# (n=6)	74.0^{a} (25.5)	0.13^{a} (0.07)	0.6^{b} (0.3)	1.6 (3.8)	1.0 (0.7)	24.7 (12.5)	0.0 (0.0)	24.7 (12.5)	0.5 (0.7)	0.18^{a} (0.07)	0.08 (0.04)
Waffles, frozen/prepared from mix# (n=5)	43.4 (39.4)	0.16^{a} (0.06)	0.4^{a} (0.4)	<0.1 (0.2)	0.4 (0.3)	19.5 (14.6)	0.0 (0.0)	19.5 (14.6)	0.9 (0.8)	0.13^{a} (0.10)	0.09^{a} (0.04)
Quick Bread, Cakes and Cookies											
Chocolate cake/cupcake (n=5)	32.8 (24.7)	0.07 (0.02)	0.21^{a} (0.12)	<0.1 (<0.1)	0.2 (0.2)	10.9 (4.5)	0.0 (0.0)	11.0 (4.5)	0.6 (0.5)	0.11^{a} (0.04)	0.05 (0.04)
White cake/cupcake (n=5)	66.7^{a}(29.4)	0.05 (0.02)	0.32^{a} (0.05)	1.1 (1.6)	1.0 (1.1)	12.0 (3.9)	0.0 (0.0)	12.0 (3.9)	0.2 (0.1)	0.12^{a} (0.01)	0.03 (0.01)

Brownies, commercial or as prepared from mix# (n=5)	40.2 (42.2)	0.08 (0.06)	0.23 (0.13)	<0.1 (<0.1)	0.9 (1.0)	10.9 (1.7)	0.0 (0.0)	10.9 (1.7)	1.6 (2.3)	0.09 (0.03)	0.05 (0.05)
Muffin, blueberry (n=5)	34.1 (15.6)	0.10[a] (0.04)	0.29[a] (0.11)	0.4 (0.7)	0.3 (0.2)	25.1[a] (26.2)	8.1 (18.2)	11.2 (5.2)	1.1[a] (0.8)	0.16[a] (0.09)	0.09[a] (0.07)
Banana bread (n=4)	27.2 (4.2)	0.13 (0.08)	0.27[a] (0.07)	2.0 (3.0)	0.2 (<0.1)	13.8 (5.9)	0.0 (0.0)	13.8 (5.9)	0.3 (0.2)	0.11 (0.02)	0.04 (0.01)
Cookies, chocolate chip (n=5)	15.7 (16.3)	0.10 (0.04)	0.17 (0.14)	2.5 (0.7)	0.1 (0.1)	9.9 (5.6)	0.0 (0.0)	9.9 (5.6)	0.3 (0.1)	0.06 (0.06)	0.02 (0.02)

* Data represent mean (standard deviation). #As prepared according to package instructions to add egg, vegetable oil or butter and milk or water. [a] Source of vitamin or mineral (>5 % DV per Health Canada reference serving). [b] High source of vitamin or mineral (>15 % DV per Health Canada reference serving). [c] Excellent source of vitamin or mineral (>25 % DV per Health Canada reference serving)
